# Supplementary material for: High Cerebrospinal Fluid CX3CL1 Levels in Alzheimer’s Disease Patients but Not in Non-Alzheimer’s Disease Dementia
Source: J Clin Med. 2022 Sep 20;11(19):5498. doi: 10.3390/jcm11195498 (PMC9571188; doi:10.3390/jcm11195498)

Supplementary

Figure S1 – Ab 1-42, Ab 1-40 and ratio Ab 42/40 values in single subjects belonging to Group A and Group B.

Figure 1S - Aβ 1-42, Aβ 1-40 and ratio Aβ 42/40 values in single subjects belonging to Group A and Group B.

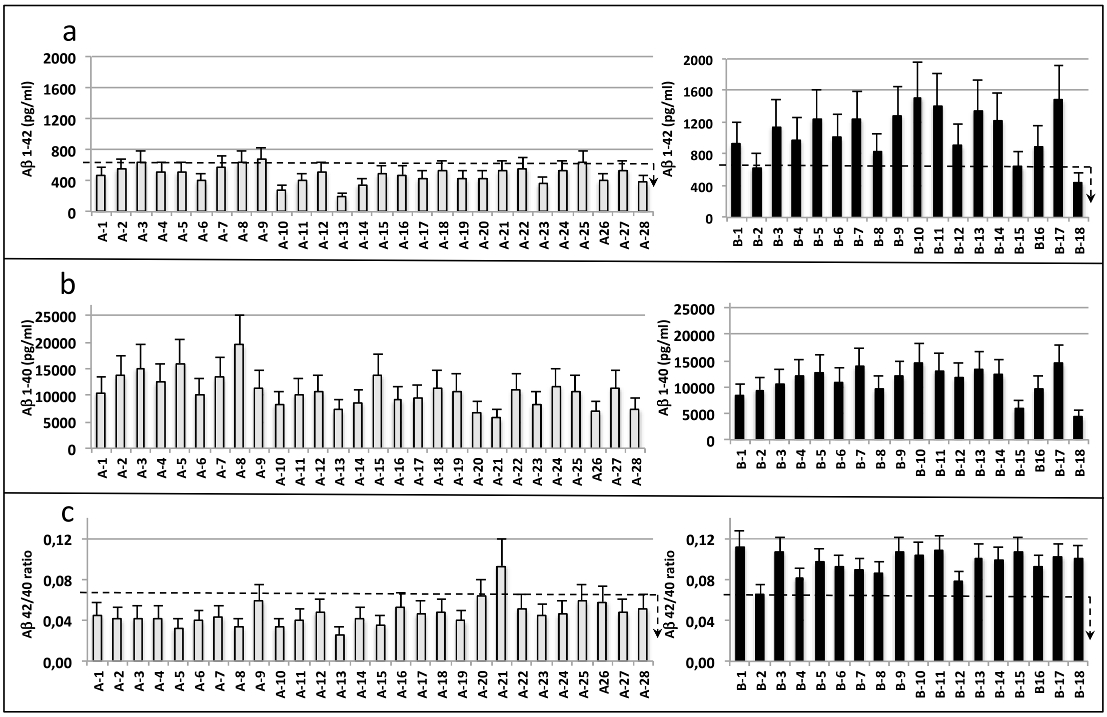

In Figure S1, the values of the different variables are reported from all investigated patients. In (a) are reported the amount of Aβ 1-42 (ng/ml) present in each analysed person. In (b) the quantity of Aβ 1-40 (ng/ml) and in (c) the ratio Aβ 1-42/1-40. In the figure, the dotted line represents the value for which a subject can be considered heath or AD; the arrow direction indicates values for which subjects are considered AD.

Figure S2– Tau-total and Tau-phosphorylated values in single subjects belonging to Group A and Group B.

Figure 2S - Tau-total and Tau-phosphorylated values in single subjects belonging to Group A and Group B.

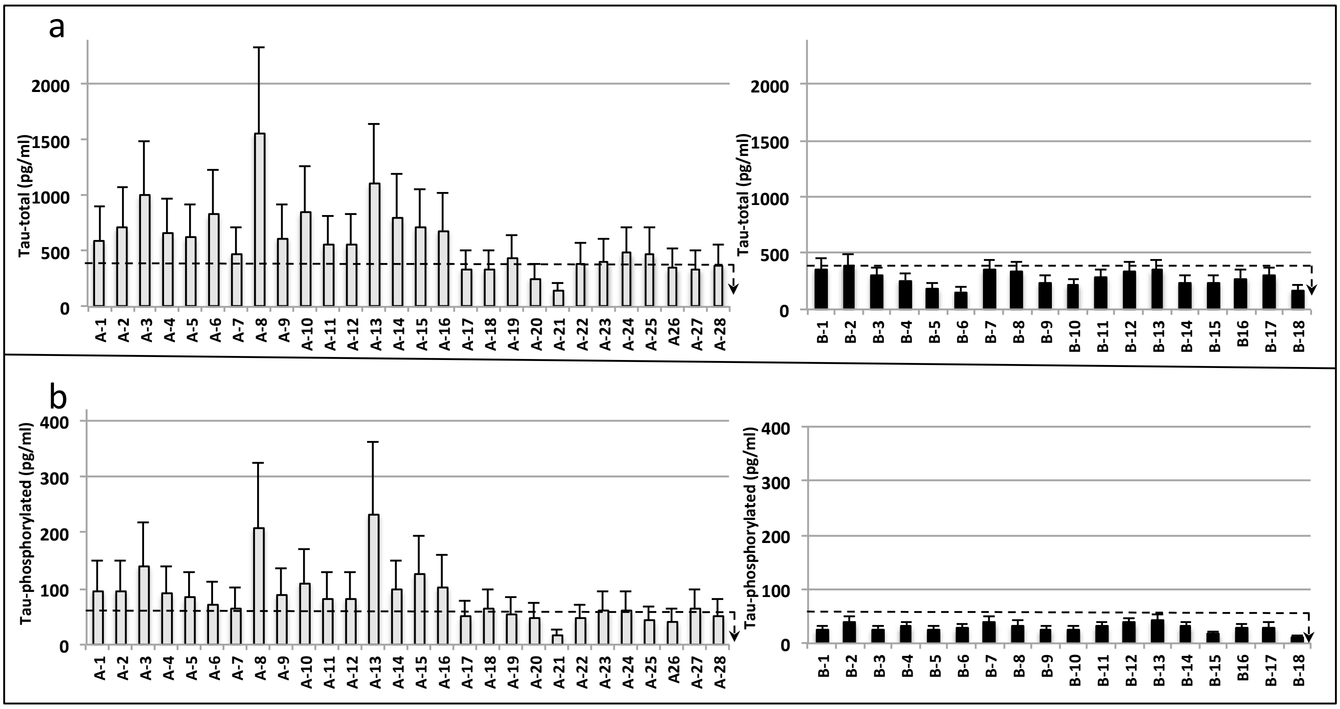

In Figure S2, the values of the different variables are reported from all investigated patients. In (a) are reported the amount of Tau-total (ng/ml) present in each analysed person. In (b) the quantity of Tau-phosphorylated (ng/ml). In the figure, the dotted line represents the value for which a subject can be considered heath or AD; the arrow direction indicates values for which subjects are not considered AD.

Figure S3 – Ab 1-42, Ab 1-40 and ratio Ab 42/40 values in single subjects belonging to Group A, divided into Group A’ and Group A”, and Group B.

Figure 3S - Aβ 1-42, Aβ 1-40 and ratio Aβ 42/40 values in single subjects belonging to Group A, divided into Group A’ and Group A”, and Group B

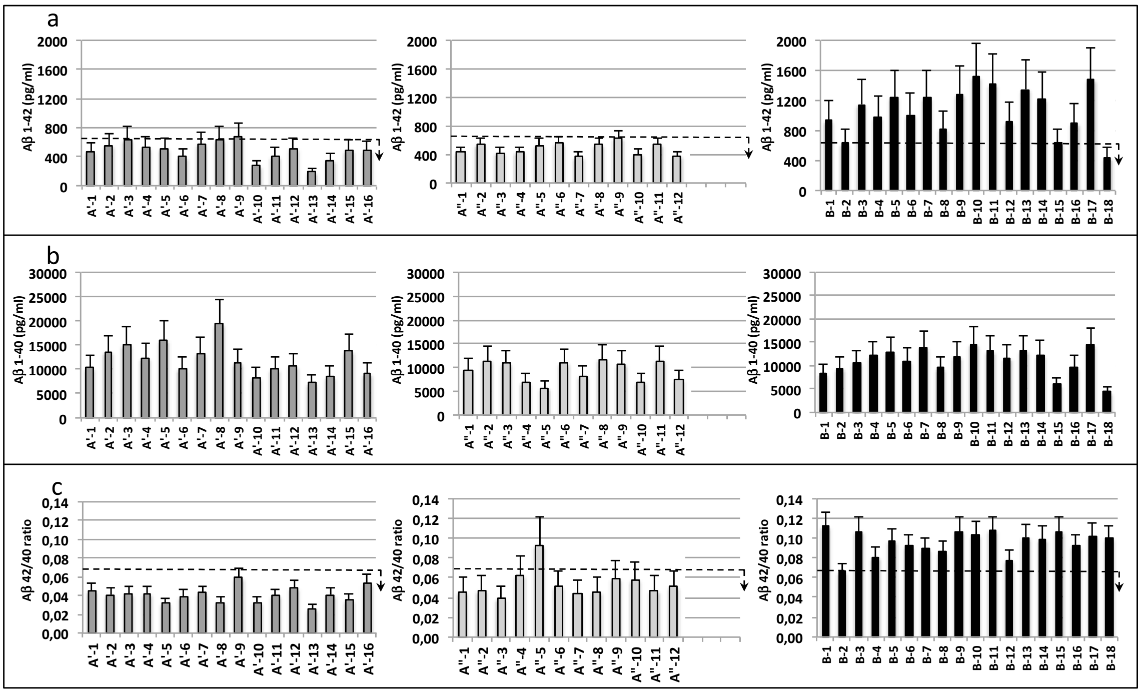

In Figure S3, the values of the different variables are reported from all investigated patients. In (a) are reported the amount of Aβ 1-42 (ng/ml) present in each analysed person, belonging to Group A’, Group A” and Group B. In (b) the quantity of Aβ 1-40 (ng/ml) and in (c) the ratio Aβ 1-42/1-40, again belonging respectively to Group A’, Group A” and Group B. In the figure, the dotted line represents the value for which a subject can be considered heath or AD; the arrow direction indicates values for which subjects are considered AD.

Figure S4– Tau-total and Tau-phosphorylated values in single subjects belonging to Group A, divided into Group A’ and Group A”, and Group B.

Figure 4S - Tau-total and Tau-phosphorylated values in single subjects belonging to Group A, divided into Group A’ and Group A”, and Group B.

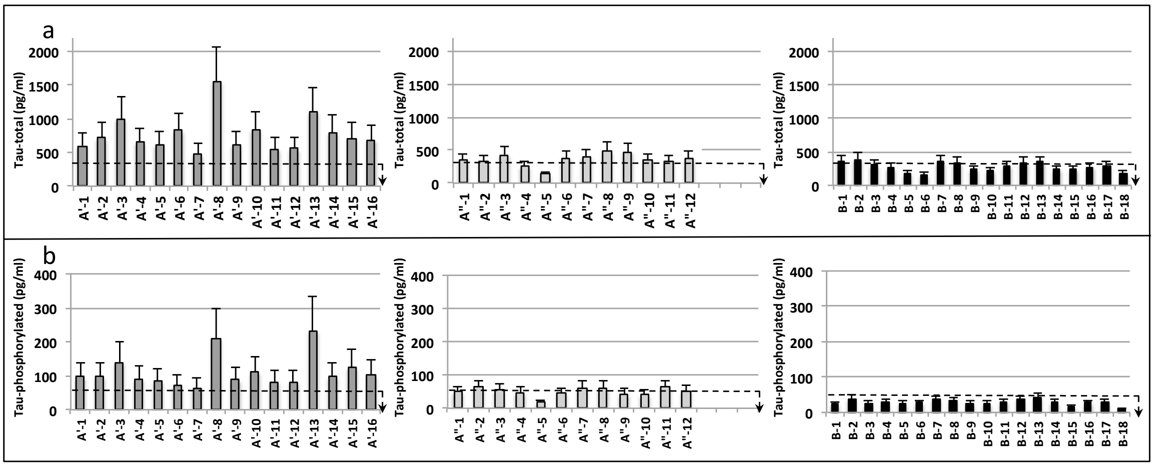

In Figure S4, the values of the different variables are reported from all investigated patients. In (a) are reported the amount of Tau-total (ng/ml) present in each analysed person, belonging to Group A’, Group A” and Group B. In (b) the quantity of Tau-phosphorylated (ng/ml) in patients belonging respectively to Group A’, Group A” and Group B. In the figure, the dotted line represents the value for which a subject can be considered heath or AD; the arrow direction indicates values for which subjects are not considered AD.

Figure S5– CX3CL1 values in subjects belonging to Group A, divided into Group A' and Group A", and Group B.

Figure 5S - CX3CL1 values in subjects belonging to Group A, divided into Group A' and Group A", and Group B.

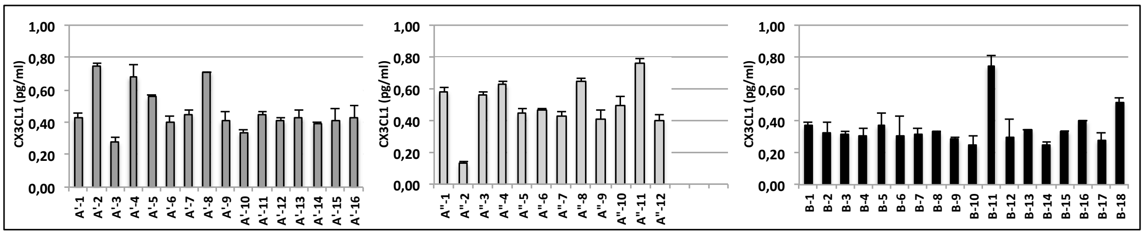

In Figure S5, the amount of CX3CL1 present into CSF of all investigated patients belonging to Group A', Group A" and Group B, are reported.

Figure S6 – CX3CL1 values in single subjects belonging to Group A and Group B.

In Figure S6, the amount of CX3CL1 present into CSF of all investigated patients, Group A and Group B, are reported.

Figure 6S - CX3CL1 values in single subjects belonging to Group A and Group B.

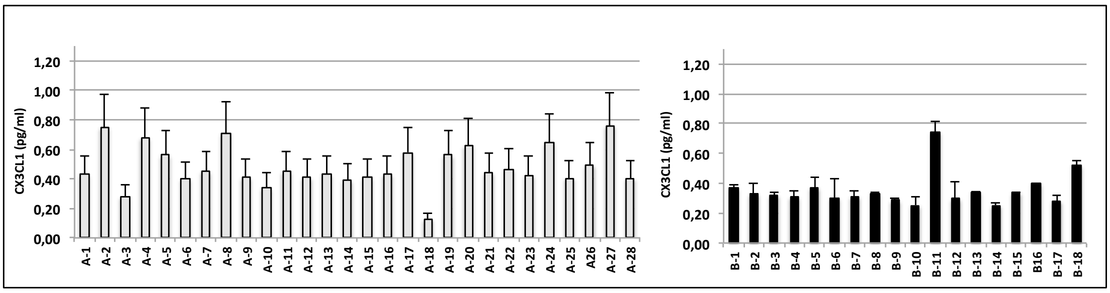

Supplement: Supplementary file 1 [file jcm-11-05498-s001.zip › jcm-1849988-supplementary.pdf]
